# A qualitative exploration of perceived challenges and opportunities in the implementation of injury prevention and management in amateur female sport

**DOI:** 10.3389/fspor.2024.1430287

**Published:** 2024-07-10

**Authors:** Ayrton Walshe, Ed Daly, Lisa Ryan

**Affiliations:** Department of Sport, Exercise and Nutrition, School of Science and Computing, Atlantic Technological University, Galway, Ireland

**Keywords:** female, qualitative, sports science, sports medicine, field-sport, injury

## Abstract

**Introduction:**

Coaches, practitioners, and leadership in amateur female sport must navigate many obstacles in the pursuit of athlete availability and optimal performance. The present study aims to evaluate opportunities and challenges to both injury prevention and management in amateur female sport, as experienced by mixed-gender coaches, allied healthcare professionals, and general practitioners.

**Methods:**

Semi-structured virtual interviews of coaches, allied healthcare professionals, and general practitioners (*N* = 25), recruited via convenience snowball sample. Data transcribed verbatim with reflexive thematic analysis through a critical realism framework.

**Results:**

Female-specific issues, education, and resource capital were challenges to the implementation of injury prevention and management in amateur female sport, thus negatively impacting on performance, prevention, and rehabilitation. Opportunities for improved care for female athletes were developing communication and relationships, outsourcing responsibility, and providing greater education, in response to desires for such, and lastly exposure to elite sport and national governing bodies’ protocols.

**Discussion:**

In amateur female sport, developing communication pathways and relationships, along with upskilling coaches, can help better support female athletes, coaches, and healthcare professionals. Leaders and stakeholders must advocate for and support greater education, resourcing, and an understanding of female-specific issues in amateur female sport. It is intended that these findings will provide evidence and opportunities for discourse between stakeholders in amateur female sport to improve standard of supports for female athletes, coaches, and healthcare professionals. These findings may also help practitioners better exploit opportunities and circumvent challenges to improve the welfare and performance of amateur female athletes.

## 1 Introduction

Female sport has experienced positive growth in participation and commercialization internationally as policymakers and governing bodies aim to improve equality of gender and address the historical male hegemony of sport (1–3). This challenge extends to sport science and medicine literature where a lack of female-specific research results in androcentric findings being implemented in female athletes (4, 5). Such obstacles also necessitate further development of female coaches to advocate and accelerate such change, but currently a lack of opportunities, gender-related issues (such as sexism or bullying), and a lack of governing body or club support commonly exist (6–8).

Despite female-specific research gaps, there is an understanding of distinct injury epidemiology and aetiology compared with for male athletes. Studies have highlighted high rates of knee, ankle, and sport-related concussions (SRCs) in female field-sport athletes (9–11). These injuries can result in significant time loss for female athletes. An SRC can take up to a month for female athletes (12, 13), while an ACL can take between 8.30 and 11.10 months for a full return to play (14). These injuries can also place financial burdens on athletes and insurance claims, supporting the need for injury prevention and management strategies in female athletes (11).

Injury prevention strategies may include appropriate warm-ups, load management, and strength and conditioning. Injury management strategies may include manual therapy, rehabilitation exercises, and graduated return to play (RTP). Both can improve athlete availability and enhance key performance indicators (KPIs) in female athletes (15, 16). These duties should ideally involve cross-disciplinary collaboration of suitably qualified strength and conditioning professionals, allied healthcare professionals (AHPs) (e.g., athletic therapists), and traditional medical professionals (e.g., general practitioners) (15, 17).

In amateur female sport, the availability of sport science and medicine professionals is limited. Qualitative research in Irish amateur female sport has found sub-standard or non-existent medical support in a number of sports, even where allied medical support may be available (18). Research in Gaelic games (native Irish sports including Gaelic football and camogie) athletes found only 11.10% of females had access to medical personnel at every training and match, while 26.10% never had access to such support (compared with 7.20% for males) (19).

The rates of strength and conditioning provision in amateur female sport are currently ambiguous. Research in the above Gaelic football and camogie cohort also found 55.70% of female athletes had no access to an athletic development coach, compared with 34.40% of male athletes (*p* < 0.01) (19). In elite international rugby union, qualitative research in strength and conditioning professionals acknowledged the lack of athletic development opportunities for their female athletes at their clubs compared with their male counterparts (20). This limited provision of professionals and resources often leads coaches and athletes to assume individual responsibility and resort to improvised injury prevention and management.

National and international governing bodies have acknowledged this challenge, developing injury prevention programmes in soccer (association football) (e.g., FIFA 11), rugby union (e.g., ENGAGE), and Gaelic games (e.g., GAA 15). These programmes have been effective in reducing non-contact injuries, in particular hamstring strain and overuse injuries (21–23). Pragmatic barriers (time, cost) often limit coach education towards such interventions to a single day, but effective implementation may be enhanced by future iterations involving greater support and feedback (24, 25). The rate at which such interventions are implemented in amateur female sport at present is also unclear (19).

Given the potential impact of injury prevention and management in amateur female sport, the following study aimed to explore the experiences of those who interact with amateur female athletes (coaches, allied healthcare professionals, and general practitioners). This exploration aimed to better understand the scope and implementation of injury prevention and management at present, and to determine the perceived challenges and opportunities of such strategies.

## 2 Materials and methods

### 2.1 Study background

This study implemented reflexive thematic analysis through critical realism. A critical realist framework was applied because of its suitability for explanatory research and the pursuit of casual relationships (26). The present study pursued a deeper understanding as to the causal relationships that encouraged or discouraged injury prevention and management in amateur female sport. This framework also allowed for explorations of depth reality, that is to say exploring reality through participants’ experiences and perception, the physical events they describe, and the mechanisms that create such scenarios (the casual relationships) (27). Reflexive thematic analysis also recognizes researcher subjectivity in the creation of meaning, thus this study has been prepared based on such experiences (28). AW is a male graduate in BSc Sport & Exercise Science and is embedded in the Irish Concussion Research Centre as a PhD student exploring and validating SRC-RTP in female sport.

The following methodology is prepared in accordance with the consolidated criteria for reporting qualitative research guidelines (COREQ), available in Supplementary Material S1. This study has also been prepared from a sister qualitative exploration of SRC management in amateur female sport (18). This study refers to females as a gendered term unless discussing physiology, in which case female sex is being referred to.

### 2.2 Sampling

Coaches of female athletes (*N* = 13), AHPs (*N* = 9), and general practitioners (GPs) (*N* = 3) were recruited; this sample included 15 men and 10 women. A convenience sample of six participants was initially recruited through email, text, or via LinkedIn (two per sub-group), with a snowball sampling approach applied thereafter whereby each participant would provide the name of another potential participant. This process was continued until data saturation was achieved (whereby no new themes emerged). Two of these participants were known to AW prior to study commencement.

Table 1 presents the summary data for each participant. To be included, coaches and AHPs were required to have a minimum of 1 year experience in coaching or providing pitch side care to female athletes. Many participants had participation, coaching, or professional experience in amateur male sport. As such, questioning was stated to be specific to their experience in female sport. No minimum requirements were placed on education as there was a desire to capture data on all persons who operate as AHPs and coaches in amateur female sport. Additional attempts to recruit GPs proved unsuccessful because of “lack of time”, and a belief that the study proposed topic of “injury experiences in female athletes” was not relevant to them. Two GPs and one AHP withdrew before interviews took place after initially agreeing to participate. The AHPs included physiotherapists, physical therapists, certified athletic therapists, and sports therapists. Coaches and practitioners worked with female athletes in rugby union, soccer, and native Irish sports within the GAA such as Gaelic football (See here), and camogie (See here). These experiences were at amateur club, provincial, and national league levels across the various sports.

**Table 1:** Education and experience of coaches (C), allied healthcare practitioners (P), and general practitioners (GP).

Code	Gender	Primary discipline	Level	Coaching/practice experience (years)	Level of education/coaching
C01	Male	Soccer	Club/collegiate	8	UEFA C licence
C02	Male	Rugby union	Club	8	IRFU level 3 performance coach
C03	Male	Gaelic football	Club/collegiate	15	GAA level 1
C04	Female	Camogie	Club/inter-county	12	GAA level 1
C05	Male	Camogie	Club/inter-County	7	GAA Foundation
C06	Male	Camogie	Club/inter-county	10	MSc SES-related area/GAA level 2
C07	Male	Camogie	Club/inter-county	18	BSc SES-related area/GAA level 2
C08	Female	Camogie	Club/inter-county	10	BSc physical education/GAA level 2
C09	Male	Gaelic football	Club/inter-county	10	BSc SES-related area/GAA level 1
C10	Male	Rugby union	Club/provincial	5	IRFU level 3 performance coach
C11	Female	Rugby union	Club/provincial	10	IRFU level 3 performance coach
C12	Female	Soccer	Club/sub-elite	10	UEFA A licence
C13	Female	Soccer	Club/sub-elite	15	BSc Physical Education/UEFA Youth A licence
P01	Female	Gaelic football	Club/inter-county	2	BSc Physio underway/level 6 sports therapy and rehab
P02	Female	Multi-sport	Club/collegiate	4	BSc Physio/BSc Medicine underway
P03	Male	Multi-sport	Club/provincial/inter-county	16	BSc SES-related area/MSc Sports Medicine
P04	Female	Gaelic football/Camogie	Club/inter-county	1	Level 6 in sports therapy and rehab
P05	Male	Multi-sport	Club/inter-county	4	BSc Athletic Therapy/MSc Physio underway
P07	Male	Multi-sport	Club/inter-county/elite	10	BSc in Physical Therapy
P08	Female	Camogie/rugby	Club/inter-county	2	BSc Athletic Therapy
P09	Male	Gaelic football/rugby	Club/collegiate	6	BSc Athletic Therapy/MSc Physio
GP01	Female	GP	N/A	20	BSc Medicine
GP02	Male	GP	N/A	30	BSc Medicine
GP03	Male	ED/GP^a^	N/A	1.5 (ED 7)^a^	GP training underway/GP diploma in MSK injury

^a^ ED, emergency department.

### 2.3 Data collection

Semi-structured interviews were conducted to allow for adaptation depending on the individual's background and experiences; such interviews also allow interviewers to gather in-depth accounts of personal experiences (29). Participants were invited to participate in an informal interview exploring “injury experiences in female athletes”. This broad line of questioning encouraged open discourse and allowed participants to guide the conversation with minimal direction. A reflexive thematic analysis approach was utilized to explore themes across the entire data set. Reflexive thematic analysis provides an opportunity to approach qualitative research with malleable, accessible, and transparent guidelines that appreciates the role of researcher subjectivity and overall knowledge production rather than discovery within analysis (30).

Ethical approval was granted by ATU's Research Ethics Sub-Committee of Academic Council and each participant completed informed consent forms prior to attending virtual interviews on Microsoft Teams. AW conducted pilot interviews with non-eligible coaches to trial the interview format and ensure the interview questions flowed succinctly. Mock interviews were also held with the research team to train AW prior to study commencement. No changes were made following pilot interviews. Interviews averaged (mean ± SD) 47.17 ± 7.5 min in total, each interview commenced with AW explaining the study purpose and interview structure, which included participant background, current experiences and practices, and improving best practice in injury treatment in Irish amateur female sport. A standardized list of interview questions was utilized, but the questions were adapted depending on how each conversation developed. Interviews were recorded on Microsoft Teams and converted to text transcripts in Microsoft Word. The transcripts were compared with the original audio to allow for syntax amendments and to anonymize data; the transcripts were also verified by each member of the research team.

### 2.4 Data analysis

**Figure 1:**
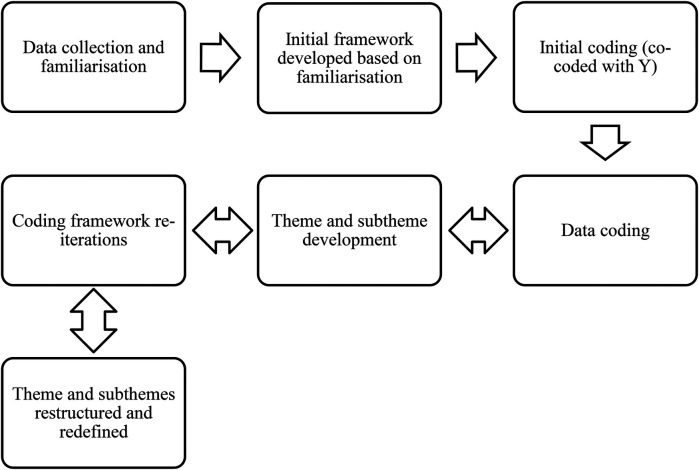
Study data analysis process following Braun and Clarke’s reflexive thematic analysis (30).

Data were analysed using reflexive thematic analysis. Semantic codes were included, but codes were primarily latent in nature to capture the underlying ideas, assumptions, and socially constructed beliefs of participants within their responses. This is ultimately necessary as critical realism is informative of reality, but not a direct reflection of it, and thus requires deeper analysis of conversations and codes (31). The transcripts were coded individually by AW in Microsoft Word (MS Corporation, USA) and later exported to Microsoft Excel (MS Corporation, USA); an initial coding framework was used to guide the coding process following familiarization with the interview transcripts. Initial interviews were also cocoded by LR to confirm agreement on the coding framework implemented; however, this framework was reflexively amended throughout the data collection process. As patterns emerged across the data set, codes were summated into sub-themes, which then formed into potential themes. These themes were redefined, revisited regularly, and their sub-themes restructured throughout the data analysis process before the final themes were summated and interwoven within shared meaning in the central organizing concept (30, 32). The data analysis process is available in Figure 1; all coding was manually completed and no coding software tools were utilized. To improve the trustworthiness of data, a number of steps were taken. A “critical friend approach” was implemented with ED and LR providing robustness, insight, and critique through data collection and analysis (33). The participants were provided the opportunity to review and amend their manuscripts to ensure accuracy of transcription and syntax; however, no such changes were required. AW also kept a reflexive diary that tracked interpretations, perceptions, and mind maps throughout the study duration to further support reflexivity in data analysis (33).

## 3 Results

Challenges and opportunities to injury prevention and management in amateur female sport were identified within participants’ interviews. Three categories and eight sub-categories are presented for each theme in Tables 2, 3, with associated quotes from participants.

**Table 2:** Challenges to implementing injury prevention and management in amateur female sport.

Theme	Category	Sub-category	Quote
Challenges to injury prevention and management (2.0)	Female-specific issues (2.1)	Injury prevention challenges in female sport (2.1.1)	C03 “Maybe sometimes with the* *…* *the senior teams or junior teams. I suppose at that level you'd have a lot of younger girls playing against girls who were a lot bigger and a lot more physical with them. That's just the nature of the grading, I suppose…particularly in X County, I find there's just huge discrepancies in terms of ages and stuff”
		Less injury prevention and management knowledge in female sport (2.1.2)	C05 “I really don't want to be disrespectful to anyone, but I do find on the ladies sports side of things* *…* *now they might be great coaches but they're a hell of a lot less educated in terms of S&C* *…* *or injury prevention”
		Sexism and inequality in female sport (2.1.3)	P02 “Like I've had* *…* *you know I've had male coaches come up to me at halftime looking for a rub on the hamstring themselves and I can guarantee they wouldn't do that if that was the male physio that was there”
	Educational challenges to injury epidemiology and treatment (2.2)	Lack of education and CPD (2.2.1)	C04 “and the knowledge I suppose of the people involved at club level, you know what they don't see, maybe strength and conditioning as a huge part of injury prevention”
		Poor understanding of female physiology (2.2.2)	GP03 “Uh knees, lot of knees, and like I don't know why knees, but the girls seem to struggle with knees”
	Lack of resources (2.3)	Time capital (2.3.1)	P02 “Like it's hard enough to get management teams for* *…* *a lot of clubs, never mind trying to train them up to be implementing all this* *…* *this extra work (injury prevention)”
		Financial capital (2.3.2)	P06 “No, to be honest with you, I wouldn't think so at all, especially in* *…* *like let's be honest, it does cost clubs a bit money to have physio and medical personnel there. You know so especially the small community clubs, maybe the GAA ones out in the countryside at times, wouldn't be able to afford it”
		Attitude capital (2.3.3)	P09 “Yes, so (smiles) coaches, what I think are barriers, coaches education. Some coaches are still…are still against S&C. Don't even mind injury prevention, you know? Like just old-fashioned ways and education and their kind of persistence with being kind of one way”

CPD, continuing professional development.

**Table 3:** Opportunities in implementing injury prevention and management in amateur female sport.

Theme	Category	Sub-category	Quote
Opportunities for injury prevention and management (3.0)	Injury knowledge and education (3.1)	Desire for education (3.1.1)	C13 “For example, on my Elite Youth A license, I was on it two months ago. And they brought in a psychologist to speak to us about like how important sleep was. And I'm just there, like this is so important. Like I wish this was on a course, four courses before that so that we're not just hitting it now at the elite level. This is actually really important for kids before the elite level”
		Access to elite sport structures and NGB protocols (3.1.2)	C04 “and again I'm lucky I suppose in the inter-county setup that the S&C Coach looks after that* *…* *the basis of a warm-up but in club teams and that, no I would do it. But I would* *…* *I pretty much copy what he'd do at times”
	Communication and relationships (3.2)	Positive communication and relationships (3.2.1)	C03 “And I’m* *…* *when I do take a team, I will* *…* *if it's an underage team, I will speak to the parents beforehand so they're fully aware of my rules when it comes to injury, when it comes to concussion, that sort of stuff”
		Desire to communicate and working relationships (3.2.2)	GP02 “I think* *…* *I think the more communication we can have between GPs and the physios, the better. You know, sometimes a consensus opinion is* *…* *is pretty good. You know that somebody should be* *…* *I'd be all for it”
		Need to communicate and build relationships (3.2.3)	P05 “if you get quite comfortable around girls and obviously it was only with certain girls you would be able to have this chat with (menstrual cycle). But they would say it themselves as like, ‘oh, this week I'm quite rundown’ and you start quizzing them and in a little bit, you'll realize that, well they're ran down for a reason. It's the week before or it's coming into that week”
	Responsibility for injury (3.3)	Ability to outsource (3.3.1)	C08 “obviously at county and everything, we are privileged. Well* *…* *I won't say we're privileged. We have the access to medical professionals there who know straight away, and they're like ‘whip them off the field’. We don't even have to think about it, they just say ‘she can't play on, concussion, suspected concussion take them off’”
		Desire to outsource (3.3.2)	C12 “like I wouldn't be comfortable making that decision or being comfortable, like happy in the knowledge that that decision is accurate or correct when I'm not a physio. Amm I would gladly *laughs* take expert advice off a physio or a doctor and take that kind of decision away from me”
		Outsourcing reducing burden (3.3.3)	C05 “I* *…* *well like I've experiences of, and this is more college level now where I had to go into games by myself and you're dreading that to be honest. You know, you're dreading that something may happen, amm and you just don't know what to do”

### 3.1 Theme 1—challenges to implementing injury prevention and management

The challenges to injury prevention and management were discussed by coaches and healthcare professionals alike in the present study, independent of the sport or playing level they operated at. These challenges included the following; female-specific issues, educational challenges, and a lack of resources (Table 2).

### 3.2 Female-specific issues

Across interviews, participants made references to a variety of challenges that were specific (or more prevalent) to female sport and the athletes they worked with. These issues were related to lesser injury prevention and management, and sexism/inequality in female sport (Category 2.1). All of these sub-categories interact and influence one another to impact female athletes.

Injury prevention challenges in amateur female sport were linked to gulfs between female athletes. Some coaches and practitioners discussed issues whereby two athletes in opposition may have had completely different physical preparation experiences within their sport, which influences injury risk and loading of the less experienced athlete. Although not a uniquely female experience, this may occur more often given the lower participation rates compared with male athletes in some sports/regions; **C08** “*if I'm an Inter-county* (Elite GAA competition) *player, and I'm going shouldering a girl who is only 17, and I'm 28, and I have had 10 years of S&C behind me, and this girl is only coming straight outta minor into adult*” (Sub-category 2.1.1–2.1.2). These gulfs may not only occur due to age or playing level but may also occur due to positive but late entry into sport. Another challenge that bears the consequences from the abovementioned issue was loading challenges in female athletes; **C05** “*You know, they wouldn't see the harm in a girl training twice in a day and maybe d*’ *you know, and maybe doing three or four sessions, dependent on a weekend*” (Sub-category 2.1.2).

Coaches also described poorer injury management strategies in female athletes. This was described by participants to be present in both coaches and athletes alike leading to poorer management strategies than their male counterparts; **C06** “*you often see that if you talk to a fella* ‘*Yeah I'm going to a* *physio tomorrow. These are the exercises I have, you know, I'm progressing well*’ *and then girls* ‘*Yeah I'm going to the physio for a rub tomorrow and hopefully it'll be OK*’” (Sub-category 2.1.2).

Lastly, challenges of inequalities and negative perceptions of female sport impacted on the experiences of those involved. Hostile sexism could be experienced by female coaches and practitioners alike; **C13** “*So like even when I moved back, like I'm very experienced. And when I moved back, it was a lot of* ‘*oh, here I'll tell you what you can do now*’*. And it was something so simple as like make sure that there's footballs around the grid.* ‘*Ah, thank you*’ *thumbs up*”. Such sexism also impacted on inequality in supports, which some teams were beginning to challenge; **C08** “*As I said, it does cost money and there was always one there for the men and I suppose it was kind of… the girls had brothers and things playing and that… They were like,* ‘*well, they're getting this. So, it's time now that we kind of follow suit and get what we should be getting*’” (Sub-category 2.1.3). If sexism is present in amateur female sport, this may have broader consequences for the equality of resourcing and representation of female coaches and professionals.

### 3.3 Educational challenges to injury epidemiology and treatment

An anticipated barrier to the uptake of injury prevention and management in amateur female sport is education (Category 2.2). Without accessible education, there is limited stimulation for the uptake of such disciplines by all parties (athletes, coaches, practitioners, and GPs), which is detrimental to effective athlete welfare protocols and overall athletic performance. Coaches and GPs lacked education and understanding in the area of musculoskeletal (MSK) injuries in particular and displayed a clear desire to improve such if provided the opportunity. In addition, poor knowledge of SRC management as advised by national governing bodies (NGBs) was also evident in the interviews; **C05** “*I suppose then it falls, you know, the management have to try and take it upon themselves, and look, they really don't have the knowledge … the time isn't there? The resources aren't there*” (Sub-category 2.2.1).

One key barrier within education was the lack of female-specific knowledge of injury epidemiology and treatment. This was particularly evident in an inability to recognize or understand the anatomical or physiological factors that result in greater risk of knee injuries in female athletes (Sub-category 2.2.2). As a result, the lack of understanding of female physiology in participants meant they regularly stated the sex of the athlete would not influence their approach to their treatment or prevention of injuries. However, in some cases participant demeanour and semantics did seem to indicate a reluctance to admit treating athletes different based on their sex in fear of this being portrayed as sexist; **C01** “*No, no, no. I've never. I've never treated anyone different based on their gender in terms of the training and the approach, I think it's, you know, it's … football is universal. Like, I think you just you, you're trying to do the same thing, doesn't matter who they I suppose.’*

### 3.4 Lack of resources

Another common barrier that consistently seemed to impact on the perceived feasibility of injury prevention and management in amateur female sport was resources and the lack thereof (Category 2.3). A lack of time, capital, education, and positive injury attitudes were the most common reason for injury prevention, injury management, and strength and conditioning not being implemented in female squads. This was particularly indicated in rural areas where all of the above was amplified to a greater extent than in clubs located in towns and cities. The following quote helps emphasize how each of these can impact progress in introducing concepts like injury prevention in amateur female sport; **C06** “*I think most clubs would be negated to this sort of stuff,* ‘*what do we need it for?*’*,* ‘*jaysus that's a bit over the top*’*,* ‘*how much money does it cost?*’ *you know so there's definitely an educational ignorance to it.* ‘*Why do we need it?*’ *You know* ‘*why … why is this fella coming in here with this idea?*’” (Sub-category 2.3.1–2.3.3).

### 3.5 Theme 2—opportunities for implementing injury prevention and management

Opportunities for injury prevention and management are evident in the present study, aiding in better procedures and care for athletes. These opportunities include the following: injury knowledge and education, communication and relationships, and responsibility for injury (Table 3).

### 3.6 Injury knowledge and education

Despite many gaps being evident in participants’ knowledge of injury prevention and management and an admittance of such in amateur female sport, there were favourable opportunities of education derived from participants (Category 3.1). For instance, across the board there was a clear desire among participants interviewed to improve their knowledge of injuries in order to better manage and support female athletes; **C04** “*On the injury prevention stuff … if there was workshops on, you know …  this warm-up reduces the chances of hamstring or ACL injuries or make sure you're incorporating this in your warm-up … I would be straight away jumping at something like that. I've seen so many ACL injuries that you'd be like, am I doing something wrong or am I not including something that I need to include?*”. This even led some of the coaches interviewed to attend higher education in pursuit of answers; **C07** “*That's why I kind of went back to X IT as well, to kind of figure out what the hell is going on with the body in women, more so than men. They're getting all these cruciate injuries, and we're not as much*” (Sub-category 3.1.1).

Furthermore, despite acknowledging the limited knowledge and implementation of injury prevention and management strategies in amateur female sport, some participants interviewed did have exposure to NGB-researched injury prevention warm-up or more generalized warm-up protocols. Coaches and practitioners seemed to value the warm-up as a key tool in their arsenal to help prevent injury and improve performance; **C11** “*So, we work off their warm-up, the injury prevention warm-up. We use that a lot and it does work, like the likes of the single leg or … very important for the knees, for females definitely*” (Sub-category 3.1.2). Although it was noted that generalized and loose descriptions (as above) were often used to describe such warm-ups, the actual translation of these protocols and understanding of such may be different in applied practice to those intended by the NGBs.

At least 16 participants in the present study had experience in both amateur and elite settings; this served as an apparent facilitator of awareness of sports science methods, in particular the need to monitor and manage training loads in athletes. While the associated technologies for such monitoring was not available in amateur sport, the coaches and practitioners could carry over this knowledge to help educate and better manage high-risk athletes within their squads; **C09** “*Take them out of certain things … you might be doing some conditioning stuff. You take them out of that* ‘*cause they wouldn't need it, you know, they'll be getting enough of that from the other sports/teams. So I … I suppose you would reduce the amount that they're doing … amount of kilometres … trying to keep them as fresh as possible*’” (Sub-category 3.1.2).

### 3.7 Communication and relationships

Positive communication and relationships are paramount to the effective manifestation of injury prevention and management in amateur female sport. Positive communication discussed by interviewees encouraged injury disclosure and management pathways to form between athletes, coaches, and members of any potential multidisciplinary team (Category 3.2). This was prominent in underage athletes where developing communication and relationships with parents’ offered an opportunity for improved disclosure and parental education on the consequences of poor injury management; **C03** “*I'll talk to parents beforehand, especially the parents of younger kids and you kinda go* ‘*look, I'll do my best not to put you … put her in a position where I think she might get hurt*’” (Sub-category 3.2.1)

It was clear from conversations that there was a desire or need to develop relationships and communicate with the various parties involved with injury management of female athletes. This was key to all parties understanding the loading of athletes each week, especially those in multiple squads or sports. Open communication and relationship development also allowed for discussion regarding the physiological health of female athletes. Only one practitioner alluded to having discourse with athletes regarding their menstrual cycle, which was dependent on rapport; **P05** “*if you get quite comfortable around girls and obviously it was only with certain girls you would be able to have this chat with (menstrual cycle). But they would say it themselves at like,* ‘*oh, this week I'm quite rundown*’ *and you start quizzing them and in a little bit, you'll realize that, well they're ran down for a reason. It's the week before or it's coming into that week*” (Sub-category 3.2.2–3.2.3).

### 3.8 Responsibility for injury management

Overall, responsibility is something that was discussed across all interviews but was perceived differently based on numerous factors such as availability of allied healthcare, academic background, culture and attitudes, and perceived competency (Category 3.3). These variations in context meant some coaches were comfortable managing injuries, some wanted parents involved—others did not, but ultimately all shared a desire to outsource this responsibility to allied healthcare where possible; **C03** “*having someone there, not that it's a … caught ya or gotcha, but you can turn around and go* ‘*Is she alright?*’ *and you're relying on someone's professional ability and that's what they're there for*” (Sub-category 3.3.1)

One caveat of responsibility and deciding where this resided was the potential for bias and improper practice influencing the decision-making processes. This links with the abovementioned communication sub-theme where some coaches held meetings with parents prior to the season commencing. These meetings facilitated clear boundaries on the decision-making responsibility for injuries such as SRCs. This in turn prevented parents from trying to overrule the coach's responsibility by attempting to allow their child to return early. From an allied healthcare point of view, each practitioner wanted to be the key instrument in the decision-making process, but also supported the inclusion of parents and coaches in the return to play process.

Many of the coaches, despite understanding the acceptance of some level of responsibility, felt burdened by the risks associated with such. This induced negative emotions when coaching, which could affect their ability to coach effectively, with some linking it to preventing athletes becoming involved in coaching within their sport; **C01** “*We didn't have a physio for example. We had this physio bag and, you know, someone got injured like or someone went down. It was very scary, you know, this is on me. I don't know anything about this. I mean you're … like, freaked out of your head*”*.* One GP empathized with first aiders and even suggested pitch side care may be a risk to their own careers; **GP01** “*But it…it would be a big ask for someone to take it on, like asking a member of the community to become like … it's a minefield even for us, you know, even to volunteer to stand at the side of the pitch. It's really controversial. I mean, will our medical insurance cover us?*” (Sub-category 3.3.2–3.3.3). While this burden may be perceived as negative, it was often this burden which encouraged coaches to pursue the provision of first aid or allied healthcare for their team.

## 4 Discussion

The present study aimed to identify challenges and opportunities for injury prevention and management in amateur female sport via the experiences of coaches, allied healthcare professionals, and general practitioners. It is intended that these findings may support coaches, professionals, and leadership in the pursuit of improved injury prevention and management of their female athletes. Through reflexive thematic analysis, the opportunities identified were as follows: access to injury education, development of positive communication and relationships, and ability to outsource responsibility. Challenges to the implementation of injury prevention and management were as follows: female-specific issues, educational challenges, and a lack of resources.

### 4.1 Overview of key findings

Across interviews, it was clear that participants believed further work is needed to support the integration of injury prevention and management in amateur female sport. Since these interviews were conducted, the GAA have launched a sport science framework that may help guide leadership towards better integration of disciplines for amateur female athletes in Gaelic football and camogie (34). Such a framework, if proven effective, could be adopted by similar NGBs to improve their own integration of sport science and hence injury prevention and management strategies. The lack of resources available was viewed as a primary limiting factor causing such issues in the present study (Category 2.3), a finding previously acknowledged in Irish female coaches (6). Lack of resources may indeed be linked to challenges such as difficulty understanding injury prevention and management and how it can impact amateur female athletes (Sub-category 2.2.1). However, it may also be affected by the investment or spread of such capital, personnel, and resources between male and female athletes seen in other countries (35–37), which has led to national and European projects being developed to improve gender equality and reduce hegemony of amateur and elite sport (38, 39). The experiences within the current study have mimicked recent qualitative research at the elite level of Irish women's soccer whereby financial and personnel restraints were leading to university students being used for medical coverage or clubs sharing pitch side medical care, which brings issues of trust into the injury treatment. This study also recognized the importance of clear communication and relationships between coaches, athletes, and AHPs to ensure injuries are fully disclosed and managed (40).

Opportunities for injury prevention and management were still present despite the challenges described above. Educational pathways and desires to interact with these were paramount to guiding the introduction of such disciplines in amateur female sport. Third level or professional education and exposure to elite sport practice helped shape and encourage any manifestation of injury prevention and management in amateur sport in coaches who had access to such. This emphasizes the need for leadership exposure and opportunities for progression of coaches in amateur female sport to be exposed to and subsequently translate even the fundamentals of injury prevention and management strategies to their amateur cohorts (6). Furthermore, having the ability to communicate and form working relationships with the various stakeholders in athlete welfare (coaches, parents, athletes, allied healthcare) provided the opportunity for informal data gathering and sharing regarding injuries, training loads, and procedures to help support better injury prevention and management of athletes.

Themes of communication have been evidenced in previous research whereby coach communication may be a predictor of concussion disclosure to coaches or sports medicine staff in collegiate athletes (41). Meanwhile research in female rugby union identified poor communication as a mediator of injury disclosure vertically (from amateur to elite programs) and horizontally (across a club program) (42). This study also found that female athletes lacked access to allied healthcare provision; this has also been observed in previous literature on female Gaelic games athletes (19, 43).

Responsibility as an opportunity to improve injury prevention and management was influenced by other sub-categories presented in Tables 2, 3. Where resources were lacking in sub-category 2.3, responsibility often fell to unqualified volunteers (i.e., coaches, parents) to manage injuries, while a coach's attitude to assuming responsibility would be further influenced by their education and experience (Sub-categories 2.2 and 3.1). The lack of provision or funding of support roles, paired with amateur coaches’ clear desire to outsource such responsibilities, presented conflict that inhibited best practice and welfare of athletes, while burdening the coaches volunteering in sport.

### 4.2 Contextualization to previous research

While novel findings do exist in the present study, the challenges of research and intervention implementation in female sport has been well established in the literature. Emmonds et al. propose a framework for evidence-based approaches in female sport that consider current literature, the female athlete, and the environment in which the athlete operates (44). Qualitative research on Ladies Gaelic football coaches and athletes exploring their injury prevention preferences indicated that interventions must prioritize education around the benefits of injury prevention and how to effectively implement such strategies (25). The present study found that while some participants had knowledge of NGB injury prevention warm-ups, there was brief, generalized language used when discussing such protocols. This may indicate that while participants knew these were useful, they may not have actually understood why and how these protocols impact injury prevention and, in some cases, simply copied what they may have seen coaches or practitioners do previously. Recent research on injury prevention programmes has also suggested that future research incorporate frameworks that support development of coach competency; however, current challenges of time and finance may limit the feasibility of such strategies without leadership advocacy and support of female coaches in this process (24).

Corrigan et al. also stated that stakeholder education was crucial to ensuring widespread uptake of and compliance with the implementation of injury prevention in female Gaelic football players (25). These conclusions were also corroborated by findings from a sister publication of the present study whereby stakeholder inaction was a limiting factor in the implementation of SRC rehabilitation protocols (18). It has been proposed that injury prevention responsibility should increase outward from children to parents, coaches, NGBs, and government bodies (45). It should be stated that the need for greater stakeholder support may not be unique to female sport. For example, a lack of support and resources has also been identified in qualitative research in Irish male field hockey athletes (46).

It is evident from current research that there is a need to develop research and education strategies in amateur female sport regarding injury prevention and management. Delphi research in female rugby union saw 86% of key stakeholders within the sport highlight injury prevention as a key research priority, with even greater priority being placed on the relationship between menstrual cycle and injury (90%), and SRC risk reduction and management (93%). The efficacy of current strength and conditioning practices in female athletes was also listed as a high priority (76%) (5).

Attempts have been made to summarize athletic performance and nutrition best practices for female athletes in Gaelic football (47) and camogie (48) that also improve injury prevention and management, but these works acknowledge that greater research is needed in this area. Previous research has identified gender-gaps in strength and conditioning provision for male and female athletes in American collegiate sport (49). Furthermore, it also found 60% of coaches working with male athletes did not believe gender should influence strength training practice, while 86% of coaches working with female athletes believed gender should have an influence on strength training. Regarding injury prevention and management, gaps in knowledge of female physiology were present among the participants interviewed. This is a pertinent point as the lack of representation of female athletes in sport science literature has led to translation of findings from male literature with little understanding of the impact on sex-differences in physiology on these methodologies (4). The treatment of female athletes similarly to males is also present in skill acquisition coaching as research in female rugby union has identified a limited, male-centred approach to tackle coaching that was perceived as the accepted norm for female participation within their sport (50). A male-centred approach to tackle technique does not take into account issues of breast injuries, which can be prevalent in female contact and collision sport athletes (51).

In the present study, participants were asked during interviews if they would individualize approaches based on athlete sex, and with the exception of one practitioner who acknowledged the menstrual cycle (Sub-category 3.2.3) the response was often “no”. Further interventions are needed to not only ascertain whether male sport science and medicine strategies transfer to female sport, but also to assess the efficacy of interventions to upskill and educate coaches and practitioners on sex-based differences in physiology and injury epidemiology.

### 4.3 Future research and practical applications

The present study has highlighted multiple avenues for future research in amateur female sport to better support coaches, professionals, and leadership in optimizing injury prevention and management of their athletes. Participants often discussed injury prevention as a broad concept with generalized connecting terms such as “warm-up”, or “recovery” with little understanding of what is attempted to be prevented and how to practically prevent it. Future injury prevention research should assess the efficacy of meshing education and competency development with injury prevention integration in amateur female coaches and athletes. The present study has also highlighted the presence of potential sexism towards female coaches and practitioners, which may be explored in subsequent research to assess the prevalence and manifestation of such instances in amateur female sport.

The practical applications of the present study are that it may inform practitioners working in amateur female sport as to the challenges and opportunities towards injury prevention and management to better aid them in addressing, circumventing, or incorporating these themes to increase adherence and best practices in their squads. This study may also act as a stimulus to encourage discourse between stakeholders, coaches, practitioners, and athletes to better develop appropriate procedures, identify areas for improvement in practice, and to ensure equality of resources to ensure male and female athletes achieve uniform access to personnel and support as required.

### 4.4 Limitations

Qualitative research requires an acceptance of inherent bias from researchers ontological and epistemological stances, which may also extend to each participants own viewpoints as they see the world. A limited sample size was included in this study and data are representative of participants current or historical experiences, therefore this cannot be representative of their entire sporting environment. Despite best efforts, it was not possible to include additional GPs in the present study. However, this may be indicative of the belief that sports medicine is not within their current scope. In conclusion, the findings of the current study emphasize that those involved in Irish amateur female sport require support in the education and implementation of injury prevention and management strategies. It is clear from the interviews conducted that access to resources, personnel, attitudes, and education were the primary drivers of encouraging or discouraging injury prevention and management.

## Data Availability

The original contributions presented in the study are included in the article/**Supplementary Material**, further inquiries can be directed to the corresponding author.
